# The effects of multifunctional MiR-122-loaded graphene-gold composites on drug-resistant liver cancer

**DOI:** 10.1186/s12951-015-0070-z

**Published:** 2015-02-12

**Authors:** Yi Yuan, Yaqin Zhang, Bin Liu, Heming Wu, Yanjun Kang, Ming Li, Xin Zeng, Nongyue He, Gen Zhang

**Affiliations:** Department of Cell Biology, Nanjing Medical University, Nanjing, 210029 China; The State Key Laboratory of Bioelectronics, Department of Biological Science and Medical Engineering, Southeast University, Nanjing, 210096 China; Institute of Stomatology, Nanjing Medical University, Nanjing, 210029 China; Department of Biochemistry and Molecular Biology, Nanjing Medical University, Nanjing, 210029 China; Department of Biomedical Engineering, Nanjing Medical University, Nanjing, 210029 China; Maternal and Child Health Institute, Nanjing Maternity and Child Health Care Hospital, Nanjing, 210029 China; Jiangnan University Medical School, Wuxi, Jiangsu 214122 China

**Keywords:** Graphene, Gold nanoparticles, Cell apoptosis, Control release, Target

## Abstract

**Background:**

Nano drugs have attracted increased attention due to their unique mode of action that offers tumor-inhibiting effects. Therefore, we have previously explored functionalized and drug-loaded graphene-gold nanocomposites that induced cancer cell apoptosis.

**Results:**

In the present study, we developed a combination of monoclonal P-glycoprotein (P-gp) antibodies, folic acid (FA) and miR-122-loaded gold nanoparticles on graphene nanocomposites (GGMPN), which promoted drug-resistant HepG2 cell apoptosis with drug targeting and controlled release properties. We also investigated related apoptosis proteins and apoptosis signal pathways by GGMPN treatment in vitro and in vivo. Moreover, we further demonstrated the inhibition of tumor growth and the apoptosis-inducing effect by means of GGMPN with a semiconductor laser in a xenograft tumor model.

**Conclusion:**

In conclusion, our results collectively suggested that GGMPN could serve as a novel therapeutic approach to control tumor cell apoptosis and growth.

## Background

Since its emergence, nanotechnology has been used in the medical field. Scientists foresee that nanotechnology has great potential in the research of cancer therapy development. Therefore, making excellent nano-material drugs with high drug loading, high targeting ability, controlled release capabilities, low toxicity, and tumor imaging functionality appears to be highly necessary [1,2].

One important component of miR-122-loaded gold nanoparticles on graphene nanocomposites (GGMPN) in this study was miRs, which were discovered in recent years as a class of 18–24 nucleotide non-coding small molecule RNA [3]. They are involved in life activities such as ontogeny regulation, cell proliferation, apoptosis and differentiation through complete or incomplete pairing of the 3′UTR, targeting gene mRNA degradation or inhibiting its translation. Various miRs related to the regulatory pathways of hepatoma cells are gradually being discovered. Many researchers have focused on differential miR expression in hepatoma cells. Expression of miR-122 is down-regulated in many hepatoma cells. As a tumor marker, miR-122 is involved in the regulation of the transfer of cancer cells in the liver and is significantly reduced or absent in liver intrahepatic metastasis. In the majority of liver cancer cells, miR-122 has low expression, and the expression level of miR-122 in the adjacent tissues is relatively high. There are differences between the expression of tumor cells and that of normal cells. The lower expression in high metastatic liver cancer cells in comparison with normal cells opens up the potential transport functionality for drug delivery [4-6].

Other important characteristics of GGMPN include the following: the gold nanoparticles can easily absorb small biological molecules and oligopeptides, lowering the chances of eliciting an immune response; the toxicity of nanomaterials is relatively low; they are not genotoxic because of their non-viral vectors; and specific molecules (such as the intracellular molecule GSH) can substitute for nucleic acid in certain concentrations [7]. The gold-gene nanoparticle vector is usually an amphiphilic substance (positive and negative electrical attraction) formed on the basis of a nano-polymer with the core-shell structure of RNA genetic drugs (miR-122). Folic acid (FA) receptor expression is highly overexpressed in cancers of epithelial origin [8]. The FA is loaded on the gold nanoparticles to provide a targeting function. The advantage of the gold gene vector is that with the effect of GSH, it can achieve controlled release of the nucleic acid, which is preferably more controllable than viral vectors and liposomes.

Graphene is characterized by its large specific surface area and can be fixed with a variety of substances, including antibody molecules, fluorescent molecules, and drugs (in this study, the P-gp antibody bound to graphene will play the role of targeting drug-resistant cancer cells) [9]. In addition, the size of graphene affects its role as a drug carrier and the effectiveness of the drug loaded on it. With regards to the selection aspect of the graphene, it should be in a range of more than 100 nm to ensure successful loading of the nano-drug onto it and to prevent it from being easily engulfed by phagocytic cells in the body. Nano-drugs can also make use of the enhanced permeability and retention (EPR) effect to locate tumor tissue [10-13].

As mentioned above, after the gold particles and miR-122 form a composite, the composite adheres onto the graphene that affects drug-resistant cancer cells (gold particles may be incorporated in the graphene). Chemotherapy is an important treatment method for liver cancer [14]. Most liver cancer patients often suffer from ineffective chemotherapy or from effective to gradually ineffective chemotherapy due to the multidrug resistance of cells [15]. Multidrug resistance occurs when tumor cells are exposed to certain anticancer drugs and evolve drug resistance; they have cross-resistance to other drugs with different structures and functions [16]. The mechanism of multidrug resistance formation is very complex, and the increased expression of the cell membrane P-gp and increased activity of GSH-transferase are two important factors [17]. The aim of this interdisciplinary study is to investigate how to use miR-122, the P-gp antibody and FA for cancer targeting, tumor imaging, and photo-thermal therapy. Size, surface charge, hydrophobicity and other surface properties will be used to enhance the killing of cancer cells by using GGMPN with a semiconductor laser. Furthermore, the toxicity profiles of the GGMPN on red blood cell (RBC) hemolysis will be explored. Finally, the mechanisms of GGMPN-induced apoptosis and cytotoxicity were also investigated [18,19].

## Methods

### Cells, animals and chemicals

The animal studies were approved by the institutional animal care and committee of Nanjing Medical University. The female nude mice (6 weeks old) were purchased from the Animal Feeding Farm of the National Institute for the Control of Pharmaceutical and Biological Products (Beijing, China). All mice were housed in the animal facility, and the animal experiments were conducted following the guidelines of the Animal Research Ethics Board of Nanjing Medical University. The animals were kept in the facility with free access to food and water. The adriamycin-resistant HepG2 cell line was purchased from the Institute of Biochemistry and Cell Biology, Shanghai Institutes for Biological Sciences, Chinese Academy of Sciences (Shanghai, China) and was cultured in RPMI 1640 supplemented with 10% FBS (GIBCO) and penicillin (100 U/mL)/streptomycin (100 mg/mL) at 37°C in a 5% CO_2_ and water-saturated atmosphere. MiR-122 was synthesized from GenePharma Biotechnology (Institute of Biochemistry and Cell Biology, Shanghai Institutes for Biological Sciences, Chinese Academy of Sciences).

### P-gp antibody-graphene oxide synthesis and characterization

Graphene oxide synthesis and collection: 1 g of graphene and 50 g of sodium chloride were milled together for 10 mins, and the polish was dissolved in water and filtered. The filtration residue was oxidized with 23 mL H_2_SO_4_ (98%) and stirred for 8 h. Then, 3 g of KMnO_4_ was gradually added to the above mixture, and the reaction temperature was maintained at 20°C. After stirring the mixture at 38°C for 30 min and then stirring again for 45 min at 70°C, 46 mL of water was added to the above-mentioned mixture and was heated to 98°C for 30 min. Then, 140 mL of water and 10 mL of H_2_O_2_ (30%) was gradually added to the above mixture. After sufficient reaction product was recovered by filtration, the filtrate was dissolved in 10 mL HCl (5%). Gradient centrifugation was used on 1 mL of the above graphene (1.5 mg/mL) to separate it by size and type. The following centrifugation conditions were used, sequentially: 2 h, 10,000 rpm/min; 2 h, 20000 rpm/min; 2 h, 30,000 rpm/min; 2 h, 40,000 rpm/min; and 2 h, 50,000 rpm/min. After centrifugation, the sample was dried at room temperature, and a JEM-2100 transmission electron microscope (TEM) was used to observe the morphology of the sample. The hydroxyl group along the 500 nm side of the graphene oxide was replaced with a carboxylic acid. Then, 5 mL of above-mentioned graphene oxide (2 mg/mL), 1.2 g NaOH (ultrasound 1 h) and 1.0 mL chloroacetic acid (Cl-CH_2_-COOH) were added to the above solution and placed in ultrasound for 2 h. P-gp antibody (1 mg/mL) was added to the above-described oxidation graphene reaction system (v/v, 1:10) to react overnight. The product was dried under vacuum at room temperature, and protein electrophoresis analysis was performed. The antibody remaining in supernatant was checked using a Protein Assay kit (Thermo Scientific, USA). The same antibody without adsorption on GGMPN was used as the control.

### MiR-122-gold nanoparticles synthesis and control relase analysis

Briefly, 0.3 mL freshly prepared sodium borohydride (0.08%), 1 mL CTAB (2.15%) and 0.32 mL chloroauric acid (1%) were added dropwise to 30 mL of a FA (1 mg/mL) solution, which was stirred for 20 min at room temperature. Gold nanoparticles mixed with 0.5 mL miR-122 (0.001 mg/mL) were stirred for 0.5 h, and the gold nanoparticles were recovered by centrifugation. The OD value of the supernatant was used to quantify the amount of miR-122 loaded. The same amount of miR-122 of the gold nanoparticles was loaded into the wells of an agarose gel to perform electrophoresis to detect GSH for miR-122 in the sustained-release experiment. For the well without GSH added, no nucleic acid showed bands. Similarly, we used miR-122 with added AO (acridine orange), which showed a fluorescence intensity change. After the disappearance of fluorescence, gold nanoparticles were added, and miR-122 was adsorbed onto the gold nanoparticles. Adding GSH (GSH replaced miR-122) enabled miR-122 to recombine with the AO. This experiment provided further evidence showing that GSH could control the release of miR-122.

Preparation and characterization of GGMPN composites: 1 mL of P-g antibody-graphene oxide (1 mg/mL) was added to the chitosan solution (1% acetic acid, pH 5.0) and sonicated for 2 h. The final product was then mixed with 0.5 mL (0.001 mg/mL) of the miR-122-gold nanoparticles and stirred overnight. The reactant from vacuum drying was GGMPN, and a JEM-2100 transmission electron microscope was used for characterization of the GGMPN morphology.

### GGMPN biocompatibility assay

A SD rat (4 weeks, orbital vein blood) RBC hemolysis experiment was used to test the blood compatibility of GGMPN. Blood was collected in heparinized tubes and centrifuged for 10 min at 1,000 g using a cold centrifuge at 4°C. Samples were washed three times with PBS. The extent of hemolysis was determined under a microscope.

### HepG2 membrane permeability and intracellular miR-122 accumulation assay

The permeability of the adriamycin-resistant HepG2 (folate receptor FR(+)) cell membrane was measured by lactate dehydrogenase (LDH CytoTox 96 assay) treated with GGMPN. The intracellular fluorescence intensity of red fluorescence-labeled miR-122 of was used to detect and quantify the accumulation of miR-122 in cancer cells with confocal fluorescence microscopy.

### SEM analysis of morphological images of HepG2 cells

HepG2 cells were seeded on top of cover glass slips; GGMPN was then administered to these HepG2 cells. After 1 h of incubation, the cells were trypsinized and fixed using 2.5% glutaraldehyde overnight. The treated cells were washed with PBS for 5 min, after which they were washed again with 30%, 50%, 70%, 80%, 90%, 95% and 100% ethanol before being carefully dried for SEM experiments.

### TEM analysis of cellular distribution of gold nanoparticles

Drug resistant HepG2 cells were treated with GGMPN, washed with PBS, and then fixed in 2.5% glutaraldehyde. Ultrathin sections were cut and mounted on copper grids. These sections were viewed under a JEOL 1200-EX transmission electron microscope equipped with energy dispersive X-ray spectroscopy (EDS). Gold nanoparticles were identified by EDS analysis, and their sub-cellular distribution was investigated from the transmission electron micrographs.

### MTT assay on cytotoxicity

Drug-resistant HepG2 cells were maintained in RPMI-1640 medium containing 10% FBS, 100 U/mL of penicillin, and 100 μg/mL of streptomycin at 37°C with 5% CO_2_ before being plated in 96-well plates (2 × 10^3^ cells/well). After overnight incubation, various concentrations of GGMPN treatment were added into specified wells. After 36 h, a 20 μL MTT solution (5 mg/mL) aliquot was added to each well. After 4 h of incubation, the supernatant was removed, and 100 μL of DMSO was added to each well. The samples were then shaken for 15 min before the optical density (OD) was checked at a wavelength of 540 nm. All experiments were performed in triplicate. Relative inhibition of cell growth was calculated as follows: Cell viability% = ([OD] test/[OD] control) × 100%.

### *In vitro* GGMPN promote cancer cell apoptosis analysis

Staining detection of DNA fragments and flow cytometry apoptosis rate determination was performed on the HepG2 cells treated with miR-122/Lipofectamine 2000 or GGMPN. Apoptotic DNA extraction of HepG2 cells was carried out by a Biovision apoptosis DNA ladder reagent and then added into an agarose gel for electrophoresis analysis.

Western blot analysis: Antibodies against the following were used: Bcl-w, Caspase 9 (mitochondrial pathway), Caspase 3, PARP, p38, and JNK MAPK (involved in cell proliferation and differentiation). The proteins were detected by enhanced chemiluminescence.

### Photothermal therapy on HepG2 cells by GGMPN

GGMPN was used for HepG2 in vitro laser hyperthermia. The laser irradiation experiment involved choosing different wavelengths of semiconductor lasers. HepG2 cells were added to the GGMPN solution, exposed to a power density of 20 W/cm^2^ of the semiconductor laser light source and irradiated for 1 min for trypan blue staining.

### GGMPN to target tumor cells image and promote apoptosis of HepG2 tumor in nude mice

HepG2 tumors in nude mice (*in vivo* model): HepG2 cells (10^6^ cells in 200 μL DMEM culture) were injected in a logarithmic growth phase in nude mice and divided into 3 groups, each consisting of 5 nude mice: the first group was the saline control group, the second group was the 1 mg/kg miR-122/Lipofectamine 2000-treated group, and the third group was the 10 mg/kg GGMPN-treated group. One week after tumor cell inoculation, when the tumor had grown to approximately 50 mm^3^ size, four groups of nude mice were injected in the tail vein with variety of drugs at 0, 2, 4, 6, 8, 10, 12, 14, 16, and 18 days. After the first 20 days, when the tumor was removed and fixed with formalin, the size of the tumor volume was calculated by the following formula: V = π/6 × [(A + B)/2]^3^, where A was the maximum diameter of the tumor and B was the minimum diameter of the tumor.

Photothermal therapy experiments in vivo: nude mice were injected in the tail vein with GGMPN. The tumor was irradiated with the semiconductor laser light source 10 times for 10 min (every two days). Then, the tumor was removed, and its final volume was calculated.

For the tumor imaging study, biodistribution activities were induced to obtain enough activity to acquire the images. GGMPN was used for confocal microscopy 3D reconstruction imaging of HepG2 cells, and the detection of green, yellow, and red separately fluorescently labeled miR-122-GGMPN in HepG2 cells was carried out. The animals were anesthetized with pentobarbital sodium intraperitoneally and were placed on the table in a side position so that the detector was positioned on the tumor region of the animal. A small animal model *in vivo* imaging instrument (Carestream Multispectral) was used (Lumina XR).

Apoptosis was achieved by terminal deoxynucleotidyl transferase-mediated dUTP nick end labeling (TUNEL) detection of DNA fragments. When observed under a microscope, dark brown cell apoptosis was found in tumor cells, while blue cells were found in normal tumor cells. Three slices of each tumor were randomly selected, and 10 images of each slice were taken for statistical analysis. Apoptosis in vivo: pictures of nude mouse tumor tissue were taken, the tumor was lysed, and protein extracts were used for western blot analysis. The antibodies used included those against Bcl-w, Caspase 9 (mitochondrial pathway), and Caspase 3 to study the relationship of the signal transduction pathway and tumor proliferation.

### Detection of gold nanoparticles in nude mice’ organs

Five mice from each group were sacrificed (carbon dioxide euthanasia) at 5 weeks to obtain organs (bone, skin, muscle, intestine, liver and tumor). The tissue was digested to measure Au levels. All of the organs were washed with distilled, deionized water and dried on paper towels. Samples were dried to constant weights at 105°C. The organs were then ground in an agate mortar and digested in aqua regia. After appropriate dilution with double-distilled H_2_O, the metal concentrations of the samples were determined by atomic absorption spectrophotometry.

### Statistical analysis

Results were presented as Mean ± SD. A t-test was performed in each group for each time point. A value of P < 0.05 was considered statistically significant.

## Results

### Synthesis and identification of GGMPN

**Figure 1:**
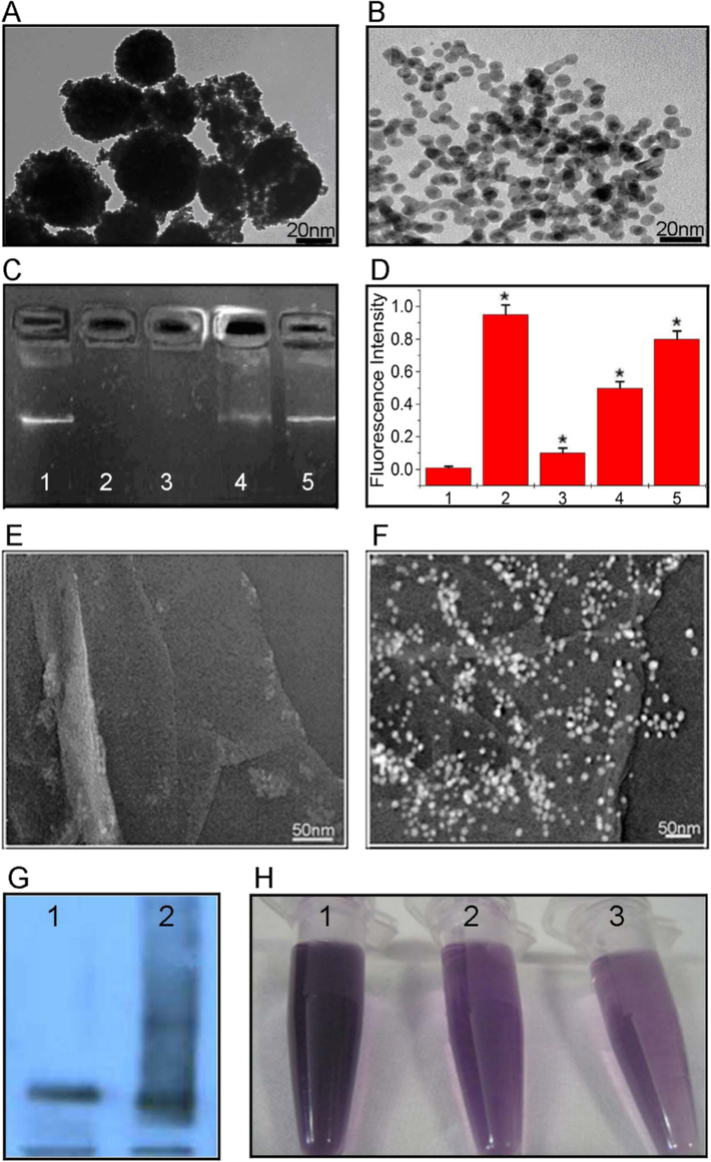
**Synthesis and characterization of miR-122-loaded graphene-gold composites. A**: HRTEM image of miR-122-loaded graphene-gold composites (Scale bar =20 nm). **B**: Low magnification image of gold nanoparticles (Scale bar =20 nm). **C**: Confirmed function of miR-122 release by GSH through agarose gel electrophoresis assay; 1) 1 mg/L miR-122, 2) 10 mg/L GGMPN, 3) 10 mg/L GGMPN + 2 μM GSH, 4) 10 mg/L GGMPN + 2 mM GSH, 5) 10 mg/L GGMPN + 10 mM GSH. **D**: Verified function of miR-122 by GSH through AO fluorescence assay; 1) AO, 2) AO + miR-122, 3) AO + miR-122 + gold nanoparticles, 4) AO + miR-122 + gold nanoparticles + 2 mM GSH, 5) AO + miR-122 + gold nanoparticle + 10 mM GSH. **E**: TEM image of graphene oxide (Scale bar =50 nm). **F**: TEM image of GGMPN nanocomplexes (Scale bar =50 nm). **G**: Protein electrophoresis analysis of P-gp antibody. P-gp antibody (lane 1), P-gp antibody-loaded GGMPN (lane 2). **H**: Quantification of P-gp antibody remaining in solution before (1) and after 1 h (2) or 24 h (3) exposure to graphene oxide.

Gold nanoparticles loaded with miR-122, termed GGMPN, were synthesized and identified using TEM imaging. We found that the complex of gold nanoparticles and miR-122 was approximately 20 nm (Figure 1A). However, the average size of the gold nanoparticles was approximately 5 nm (Figure 1B). Thus, we speculated that an abundant amount of miR-122 could be combined with the gold nanoparticles. We already knew that some small molecules (e.g., RNA) could be flexibly released by GSH from the surface of the nanoparticles. We combined negatively charged miR-122 with positively charged gold nanoparticles. As shown in Figure 1, the release reactions between gold nanoparticles and miR-122 could be performed through electrophoresis assays (Figure 1C). We demonstrated that gold nanoparticles completely prevented miR-122 from moving to the positive electrode; thus, the gold nanoparticles/miR-122 remained in the sample well (Figure 1C, lane 2). It could be observed that the positively charged gold nanoparticles counteracted the negative charges of miR-122. As expected, a small amount of miR-122 was detected at the same site of pure miR-122 (Figure 1C, lane 1) when the concentration of GSH reached 2 mM (Figure 1C, lane 4). The mobility of miR-122 completely recovered when the final concentration of GSH reached 10 mM (Figure 1C, lane 5). Negatively charged GSH contained a thiol ligand, which had a stronger affinity to the gold nanoparticles. Herein, it was established that the addition of GSH might counteract the positive charge of gold nanoparticles to some extent by place exchange and resulted in the release of miR-122 from the gold nanoparticles (Figure 1D).

As shown in Figure 1E and F, graphene oxide and GGMPN nanocomposites (500 nm) were synthesized and characterized. The TEM image demonstrated a homogeneous distribution of gold nanoparticles on the P-gp antibody-graphene oxide surface with chitosan functionality. Moreover, we also illustrated that the P-gp antibody could be effectively absorbed by graphene oxide (Figure 1H) and separated from GGMPN (Figure 1G). The results suggested that P-gp antibody-graphene oxide and GSH might play a critical role in combining miR-122 with GGMPN to enhance the targeting of miR-122 to cancer cells. The relevant miR-122 loading efficiency was further determined by OD analysis, which indicated that the miR-122 loading onto GGMPN was approximately 10%.

### Biocompatibility and cellular distribution analysis of GGMPN

**Figure 2:**
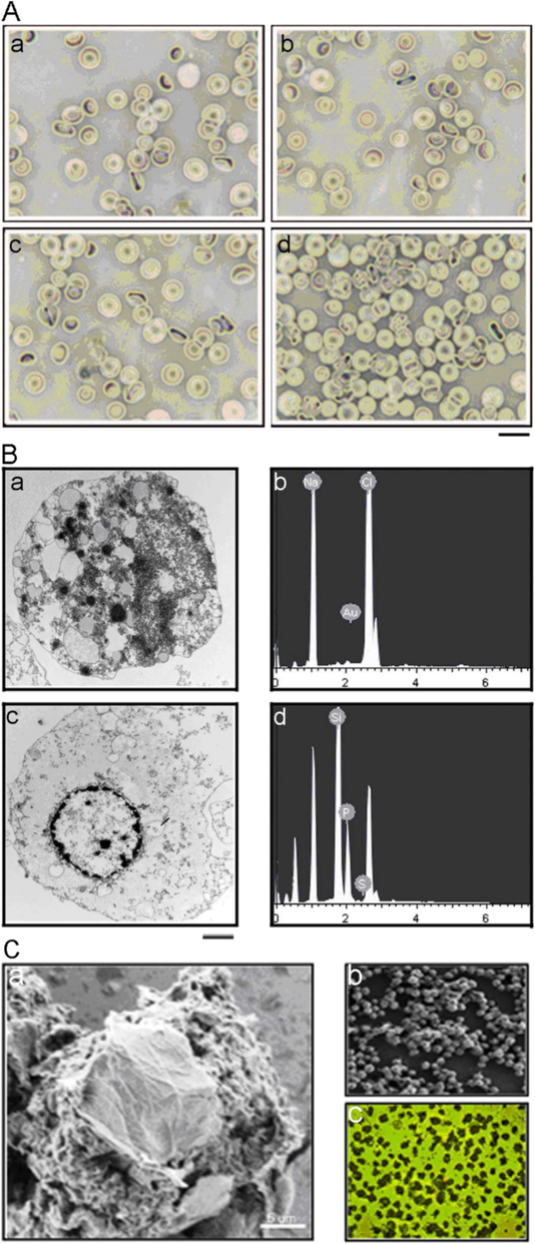
**Biocompatibility assay and TEM image of the cellular distribution of GGMPN. A:** Photographs of RBC suspensions in the presence of various reagents; **a)** 10 mg/L GGMPN for 1 h, **b)** 1 mg/L miR-122 (same concentration as loaded on GGMPN) was transfected with Lipofectamine 2000 for 1 h, **c)** untreated control, **d)** treated with 10 mg/L GGMPN for 5 h (Scale bar =20 μm). **B**: Characterization of sub-cellular distribution of gold nanoparticles by TEM and EDS; **a**, **c)** TEM image of gold nanoparticles in tumor cells treated with or without 10 mg/L GGMPN, **b**, **d)** EDS analysis of gold nanoparticles distributed in tumor cells treated with or without 10 mg/L GGMPN ( Scale bar =50 nm). **C**: **a)** SEM image of HepG2 cells after GGMPN treatment (during 1 h); **b)** SEM image of HepG2 cells transfected with miR-122 (1 mg/L, the same concentration as loaded on GGMNP) (during 1 h); **c)** Morphology of HepG2 cells after GGMPN treatments by microscope assays.

We next assessed the hemolytic effect of GGMPN in cells subjected to various treatments. For RBCs (red blood cells) treated with 10 mg/L GGMPN for 5 h (Figure 2A, d), there was no hemolysis of RBCs co-cultured with 10 mg/L GGMPN for 1 h (Figure 2A, a), of those transfected with miR-122 (Figure 2A, b), or of the untreated control (Figure 2A, b). Furthermore, it was expected that slight hemolysis would occur when RBCs were treated with GGMPN for 5 h (Figure 2A, d). The results showed that GGMPN had a low hemolytic effect when treated with cells for 5 h.

We next observed the morphological changes on sub-HepG2 cells after treatment with or without GGMPN. TEM characterization showed the typical distribution of gold nanoparticles in HepG2 cells treated with GGMPN (Figure 2B, a) compared with the untreated control (Figure 2B, c). Meanwhile, both dispersive nanoparticles and clusters of gold nanoparticles were observed in tumor cells (Figure 2B, a). Additionally, the results (Figure 2B, b) of EDS analysis remarkably demonstrated gold content in the cells, which was consistent with the above TEM study (Figure 2B, a), suggesting that gold nanoparticles could be readily internalized by cells for drug delivery. In contrast, the images (Figure 2B, d) from EDS analysis showed no gold content in HepG2 cells (not treated with GGMPN). These results suggested that GGMPN was biologically safe and could deliver miR-122 into targeted cancer cells.

Based on the above study, bio-imaging of GGMPN or miR-122 alone in HepG2 cell lines was performed with a scanning electron microscope (SEM). As shown in Figure 2C, a, the morphology of HepG2 cells was changed when treated with GGMPN. The treatment also caused stronger adsorption of GGMPN on the cell membrane compared with miR-122 transfected with Lipofectamine 2000 (Figure 2C, b). GGMPN induced the morphological changes in HepG2 cells (Figure 2C, c).

### SEM image and intracellular miR-122 accumulation assay

**Figure 3:**
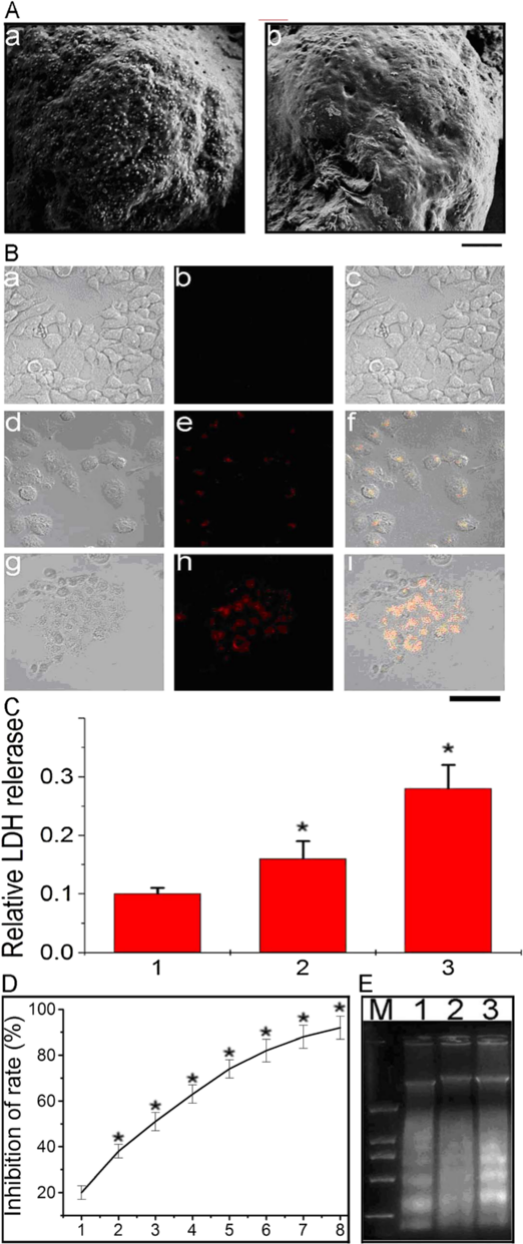
**Morphological and intracellular miR-122 accumulation assay. A**: SEM image of HepG2 cells incubated with 10 mg/L GGMPN **(a)**, or without treatment **(b)** for 1 h (Scale bar =3 μm). **B**: GGMPN delivered miR-122 into resistant HepG2 cells as imaged through laser confocal fluorescence microscopy; **a-c)** untreated cells, **d-f)** cells transfected with red fluorescent-modified miR-122 (1 mg/L, same concentration as loaded on GGMPN), **g-i)** cells combined with 10 mg/L GGMPN-loading red fluorescent-modified miR-122 (Scale bar = 50 μm). **C**: Quantitative assay of GGMPN on cell membrane permeability based on the CytoTox 96 assays; 1) untreated control, 2) resistant HepG2 cells transfected with miR-122 (1 mg/L, same concentration as loaded on GGMPN), 3) resistant HepG2 cells incubated with 10 m/L GGMPN. **D**: MTT assay for evaluation of the growth of cells treated with GGMPN. HepG2 cells were treated with 0.001, 0.01, 0.1, 1, 10, 100, 1000, or 10000 mg/L of GGMPN. *P < 0.05 indicates a significant difference in comparison to untreated control. E: DNA fragmentation of HepG2 cells after different treatments. Genomic DNA was isolated from HepG2 cells, which were treated as follows: 5 mg/L GGMPN (lane 1), 2 mg/L GGMPN (lane 2), or 10 mg/L GGMPN (lane 3); DNA marker (M).

FA on miR-122 of gold nanoparticles caused more miR-122 adherence with the gold nanoparticles on the surface of the cell membranes (Figure 3A, a) compared with cancer cells (unloaded FA on gold nanoparticles) (Figure 3A, b). We demonstrated that the miR-122 on the gold nanoparticles could precisely target the cancer cells with the FA functionality.

Next, we further determined the location of GGMPN in HepG2 cells with laser confocal fluorescence microscopy. For the control cells without treatment, we observed almost no intracellular fluorescence in the HepG2 cells (Figure 3B, a,b,c). However, the intracellular fluorescence in HepG2 cells increased dramatically upon treatment with GGMPN containing the red fluorescent-modified miR-122 (Figure 3B, g,h,i) compared with miR-122-transfected cells (Figure 3B, d,e,f). The results indicate that the intracellular content of miR-122 increased after treatment with GGMPN. These results demonstrated that GGMPN carrying miR-122 could be effectively taken in by drug-resistant HepG2 cells. They also showed that GGMPN had an impact on cell permeability compared with cells transfected with miR-122 (Figure 3C). Moreover, when increasing the concentration of GGMPN, we also demonstrated that the growth of HepG2 cells was strongly suppressed (Figure 3D), and the intensity of fragmented chromosomal DNA bands of treated HepG2 cells became much stronger (Figure 3E).

### Analysis of apoptosis in GGMPN treated cells

**Figure 4:**
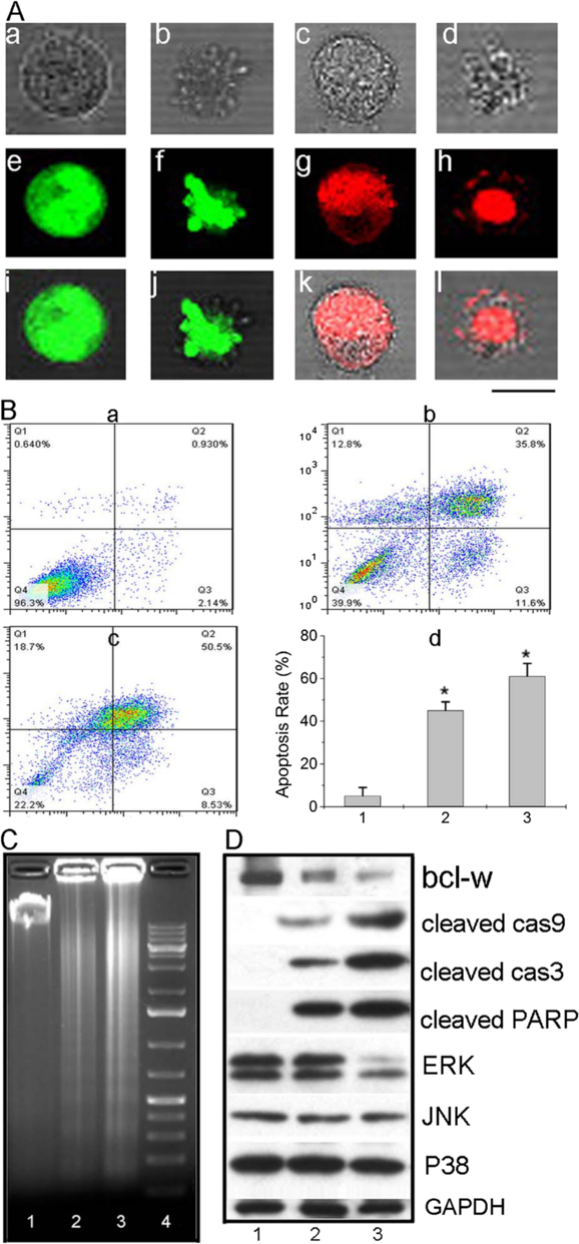
**Apoptotic assay of HepG2 cells induced by GGMPN. A**: HepG2 cells were treated with 10 mg/L GGMPN, and their apoptotic level was detected by AO/EB staining; **(a, e, i)** image of normal cells, **(b, f, j)** image of early apoptotic cells, **(c, g, k)** normal dead cells, **(d, h, l)** image of late apoptotic cells (Scale bar =10 μm). **B**: Flow cytometric measurement of cellular apoptosis of resistant HepG2 cells treated with various reagents; **a)** untreated control, **b)** transfected cells with miR-122 (1 mg/L, same concentration as loaded on GGMPN), **c)** treatment of cells with 10 mg/L GGMPN for 36 h, **d)** quantitative analysis of apoptotic cells after various treatments shown in a), b) and c), *P < 0.05, compared with the control treatment. **C**: DNA fragmentation in resistant HepG2 cells after different treatments; 1) untreated cells, 2) transfected cells with miR-122 (1 mg/L, same concentration as loaded on GGMPN), 3) treatment of cells with 10 mg/L GGMPN, 4) DNA marker. **D**: Western blot analysis of activated Caspase levels after various treatments; 1) without treatment cells, 2) cells transfected with miR-122, 3) cells treated with GGMPN.

In order to further determine the apoptotic effect of GGMPN in HepG2 cells, the AO/EB staining assay was used. Apoptotic nuclei were identified by their characteristic features such as chromosomal condensation, with distinct margination and fragmentation under fluorescence microscopy. In the present study, we also found that the apoptotic nuclei of HepG2 cells (Figure 4A (d,h,l), later apoptosis nuclei) treated with GGMPN for 48 h could be clearly identified by their distinctively marginated and fragmented appearance compared with early stage of apoptosis in cells treated for 24 h (Figure 4A (b,f,j), early apoptosis nuclei) and normal dead cells (Figure 4A (c,g,k)). For the control cells without treatment, cell nuclei were normal, as shown in Figure 4A (a,e,i).

It was shown using Annexin-V-FITC apoptosis detection that GGMPN induced a much higher apoptosis rate in resistant HepG2 cells (Figure 4B, c) than that of cells transfected with miR-122 (Figure 4B, b) or the untreated control (Figure 4B, a). Meanwhile, we also found that the percentage of apoptotic cells was 60.1%, 47.8%, and 5% for those treated with GGMPN, miR-122 and no treatment, respectively (Figure 4B, d). Furthermore, we further confirmed the apoptosis of cells induced by GGMPN treatment using a DNA fragmentation assay. When HepG2 cells were treated with GGMPN, the intensity of fragmented chromosomal DNA bands (Figure 4C, lane 3) was much higher than that observed from cells treated with miR-122 (Figure 4C, lane 2) or the untreated control (Figure 4C, lane 1).

In order to explore the molecular mechanisms underlying the GGMPN-induced DNA fragmentation, we examined the expression of apoptosis-related proteins in the cells. As shown in Figure 4D, we found that the protein level of Bcl-w, which was a target gene of miR-122, was reduced in HepG2 cells after treatment with GGMPN. Moreover, the cleaved Caspase 9 signals were much stronger in cells treated with miR-122 (Figure 4D, lane 2) than in the untreated control cells (Figure 4D, lane 1). The strongest activation of Caspase 9 occurred after GGMPN treatment (Figure 4D, lane 3). Similar results were obtained for cleaved Caspase 3 and cleaved PARP because they are the downstream elements of the Caspase 9 pathway. The MAPK signal was weaker after GGMPN treatment than after miR-122 treatment as a result of the drug-resistant nature of the cells (Figure 4D, lanes 3 and 2, respectively). The same trend of protein expression was not obtained for P38 and JNK. These data suggested that GGMPN treatment involved the inhibition of activation of anti-apoptosis proteins and caused apoptosis by activation of the Caspase-9, Caspase-3 and MAPK pathways in resistant HepG2 cells.

### Photothermal therapy on HepG2 cells treated with GGMPN

**Figure 5:**
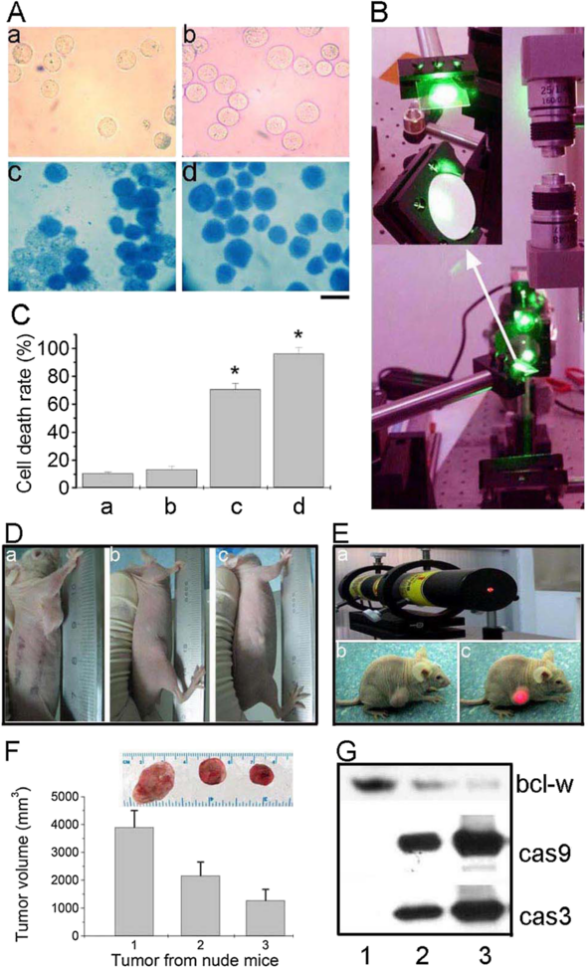
**Photothermal therapy assay of HepG2 cells treated with GGMPN. A**: Images of photothermal therapy of cells with various treatments; **a)** control experiment, **b)** transfected with miR-122, **c)** treated 10 mg/L GGMPN with laser power threshold of 20 W/cm^2^ for 1 min, **d)** or 10 min. (Scale bar = 20 μm). **B**: Photo of photothermal therapy with semiconductor laser light. **C**: Quantitative analysis of apoptotic cells after various treatments as shown in **A**, * P < 0.05, compared to the control treatment. **D**: Laser hyperthermia with laser irradiation light for inhibition of tumor growth in HepG2 nude mice with different treatments, **a)** untreated used as control, **b)** transfected with miR-122 (1 mg/kg, same concentration as loaded on GGMPN), **c)** treated 10 mg/L GGMPN with laser power threshold of 2 W/cm^2^. **E**: Photo of tumor irradiated for by laser hyperthermia; **a)** photo of laser hyperthermia, **b)** untreated control, **c)** tumor irradiated by laser hyperthermia. **F**: Quantitative analysis of tumor volume after various treatments as in **(D)**. **G**: Western blot analysis of apoptosis-related protein in tumors of mice treated as in **(D)**.

To further explore the multifunctional anti-cancer effect of HepG2 cells treated with GGMPN, we probed the GGMPN with a semiconductor laser to perform a hyperthermia experiment. As shown in Figure 5, we found that HepG2 cells treated with GGMPN were severely damaged at a laser power threshold of 20 W/cm^2^ for 10 min (Figure 5A, d) compared with other treatments (Figure 5A, a,b,c). However, no photo-thermal destruction was observed for HepG2 cells treated with miR-122 (Figure 5A, b). Quantitative analysis also showed that the percentages of dyed cells (blue cells) were 2% (control), 3.1% (miR-122 treated), 77.4% (10 mg/L GGMPN at a laser power threshold of 20 W/cm^2^ for 1 min), and 95.8% (for 10 min) (Figure 5C).

To further identify the function of GGMPN in vivo, we established a method to estimate the effect of GGMPN in tumor tissue (Figure 5D-G). In this experiment, 6-week-old mice were subcutaneously implanted with 10^6^ HepG2 tumor cells. After 10 days post-implantation, the mice were divided to 3 groups with 5 mice in each group (Figure 5D). The mice were intravenously injected with various reagents every other day. Combined GGMPN with a 2 W/cm^2^ power semiconductor laser for 10 min every two days (Figure 5E, c) effectively reduced the size and volume (Figure 5D, c) (Figure 5F, 3) of the implanted tumors compared with the untreated group (Figure 5D, a) (Figure 5F, 1) or the miR-122 treatment group (Figure 5D, b) (Figure 5F, 2).

We then further examined the effects of treatment with GGMPN on apoptotic signals. In this experiment, we examined the expression of apoptosis-related proteins in mouse-implanted tumors. As shown in Figure 5G, we found that the protein level of Bcl-w was reduced in mice tumors after treatment with GGMPN (lane 3) compared with miR-122 (lane 2) or the untreated group (lane 1). Moreover, the cleaved Caspase 9 and 3 signals were much stronger in tumors treated with GGMPN with photothermal therapy (lane 3) than with miR-122 (lane 2) or the untreated group (lane 1). These results further provided evidence that GGMPN with photothermal therapy could reduce the apoptosis and inhibit the growth of tumor *in vivo*.

### Analysis of target tumor cell image *in vivo*

**Figure 6:**
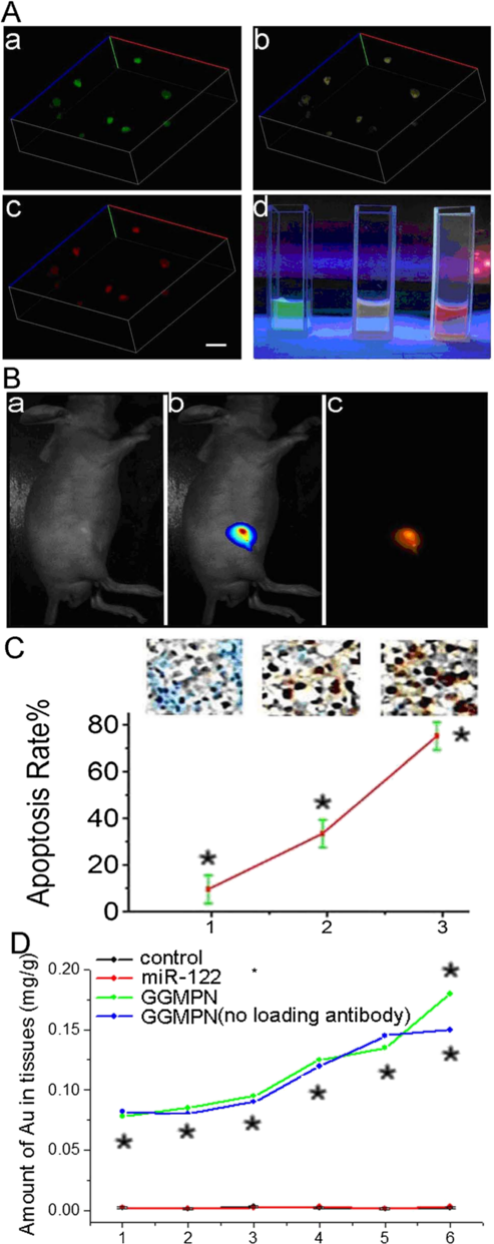
**Images of tumor cells in vitro and** ***in vivo*** **. A**: 3D reconstruction of HepG2 cells treated with 10 mg/L GGMPN with green **(a)**, yellow **(b)**, and red **(c)** fluorescence separately to analyze intracellular distribution (Scale bar = 100 μm), image of HepG2 cells treated with GGMPN labeled with green, yellow and red fluorescence **(d)**. **B**: Fluorescence image of tumor after intravenous injection of 10 mg/L GGMPN (miR-122 with red fluorescent) solution for 2 h; **a)** bright mouse image, **b)** fluorescence intensity scan of xenograft tumor, **c)** red fluorescence near the tumor. **C**: TUNEL staining of HepG2 xenograft tumors after various treatments; 1) untreated control, 2) transfected cells with miR-122, 3) treatment cells with GGMPN, quantitative analysis of apoptotic rate after various treatments **(C)**. **D**: Distribution of Au levels in the liver, the brain, the bone, the muscle, the intestines, and the tumor, respectively. The group numbers for the mouse organs are as follows: (1) bone, (2) skin, (3) muscle, (4) intestine, (5) liver and (6) tumor.

Finally, we detected the fluorescent effect of GGMPN labeled by different fluorescent dyes in vitro and in vivo. As shown in Figure 6, 3D reconstruction of HepG2 cells with GGMPN treatment demonstrated a higher intracellular distribution and target function of miR-122 (Figure 6A). Six-week-old nude mice were subcutaneously implanted with 10^6^ HepG2 tumor cells that were treated with labeled GGMPN. We found that the tumors of mice treated with labeled GGMPN could produce fluorescence spontaneously (Figure 6B, b and c). We also found that the percentage of apoptotic cells was 75.9%, 39.7% and 9.1% for treatment with 10 mg/kg GGMPN, miR-122 (transfected cells with miR-122) and the untreated control, respectively (Figure 6C). These results further indicated that GGMPN was good carrier to effectively deliver miR-122 into cancer cells. Moreover, Au concentration in nude mice injected with GGMPN was significantly higher in tumor tissues than in the other groups, as shown in Figure 6D.

## Discussion

As a novel delivery tool, gold nanoparticles (positively charged) could easily absorb small biological molecules (FA) and non-coding RNAs (miR-122). The rationale was that gold nanoparticles, FA and miR-122 were amphiphilic substances (both positive and negative electrical attraction) formed on the core-shell structure of the FA and miR-122 genetic drugs (Figure 1). Gold nanoparticles-miR-122 were formed in nanocomposites, which were further adhered onto graphene. P-gp antibody combined with graphene (Figure 1G,H) performed the targeting of drug-resistant HepG2 cells (Figure 2C). In reality, the size of the graphene is a factor that affects its functioning as a drug carrier. If the size of graphene is too large, it will affect the drug transport in the blood stream. If it is too small, it will affect the drug loading and be easily phagocytosed. Thus, we concluded that the size of graphene should be in the range of approximately 500 nm (through gradient centrifugation) to ensure the graphene drug loading and to avoid phagocytosis by phagocytic cells in vivo. As mentioned above, the nano-drug size range could use the enhanced permeability and retention (EPR) effect to target the tumor.

GSH could control the miR-122 release from the gold nanoparticles by replacing the function, which allowed the realization of sustained, controlled release of miR-122 in cancer cells. As a result of our study, the concentration of GSH in erythrocytes was 2 mM, while it was 10 mM in the resistant HepG2 cells (Figure 1C). FR was highly overexpressed in the HepG2 cell line that we selected. Due to the P-gp antibody and FA, the GGMPN could target the HepG2 cell membrane. Cellular uptake of FA is mediated by both the membrane FR and the reduced folate carrier [20,21]. In this study, we targeted cancer cells via two pathways. As mentioned above, gold nanoparticles loaded with miR-122 were formed as nanocomposites, and the nanocomposite was adhered onto graphene to induce the apoptosis of drug-resistant cancer cells (gold nanoparticles incorporated in the graphene with chitosan activity). Therefore, the nanoparticles could enter the cell by absorbing and puncturing the cancer cell membrane when miR-122-loaded gold nanoparticles were absorbed by HepG2 cells (Figure 3A). The GSH effect provided a potential environment for miR-122 entry into HepG2 cells. In fact, we selected miR-122 instead of chemical drugs for two reasons: to avoid the high toxicity of chemical drugs and to attempt to avoid the multi-drug resistance mechanism of hepatocellular carcinoma in chemotherapy.

HepG2 membrane permeability and intracellular miR-122 accumulation assays were performed. After GGMPN treatment, an increase in the intracellular intensity of the amount of miR-122 was observed in the HepG2 cells. The results demonstrated that cell membrane permeability was significantly increased by GGMPN that then induced the intake of miR-122 in HepG2 cells (Figure 3B). Consistent with this result, gold nanoparticles were also found in the HepG2 cells by TEM characterization and EDS analysis (Figure 2B). These results suggested that gold nanoparticles could be readily internalized by cells for drug delivery.

Next, we further investigated the apoptotic effect of GGMPN on cancer cells by the use of an MTT assay, nuclei staining, and a DNA fragment assay. The results demonstrated that GGMPN elicited an anti-proliferative effect in a dose-dependent manner in HepG2 cells. The apparent IC50 value for GGMPN was estimated as 10 mg/L for HepG2 cells (Figure 3D). Using AO/EB staining for apoptotic cells, apoptotic nuclei were identified by their distinctively marginated and fragmented appearance. The apoptotic nuclei of HepG2 cells (Figure 4A) at 48 h could be identified by their distinctively marginated and fragmented appearance. For the control cells without treatment, cell nuclei were normal. In effect, this was the reason why DNA extracting methods were used to produce apoptosis DNA ladders. Next, it was determined whether cell growth inhibition was caused by the apoptotic response, and the results in Figure 4B show that relevant GGMPN induced a much higher cell apoptosis rate than the untreated control using the Annexin-V-FITC, PI apoptosis detection method. The apoptosis DNA ladders were examined by agarose gel electrophoresis. HepG2 cells were treated with GGMPN, and the intensity of the apoptosis DNA ladders were much higher than that observed from untreated cells or those treated with miR-122. Our observations supported the hypothesis that the remarkable enhancement of apoptosis was induced by the synergistic effect of GGMPN. We carried out anti-cancer research on the GGMPN apoptosis signaling pathway. According to previous studies, miR-122 could induce apoptosis through the Bcl-w pathway. However, the underlying molecular mechanisms of graphene-gold materials and miR-122-induced cancer cell apoptosis were still not very clear. This study highlighted the mechanism of apoptosis in drug-resistant HepG2 cells by GGMPN treatment. We found that GGMPN treatment activates Bcl-w and Caspase 9 pathways to induce apoptosis in HepG2 cells. Cleaved Caspase-9 activated Caspase-3, which correlated with the increased cleaved PARP expression after GGMPN treatments (Figure 4D). Apoptosis DNA ladders were induced during the cell apoptosis by cleaved PARP expression.

Analysis of fluorescent bio-images of the tumors indicated that GGMPN enhanced the treated group, and the image showed features of HepG2 tumors in vivo (Figure 6B). This was expected to improve target volume consistency for treatment. The image results also indicated evidence of photothermal activity. Photothermal activity could deposit a sufficient amount of energy into the tissue under appropriate conditions to raise the temperature above a certain threshold so that cellular cancer destruction could occur. The GGMPN-enhanced photothermal killing of tumor cells by laser had been proven to be effective both in vitro and in vivo. As shown in Figure 5, cancer cells treated with GGMPN were destroyed with the laser, and the growth of the tumor was suppressed. This result was indeed consistent with the earlier observations regarding laser treatment for photothermal therapy in vitro. Meanwhile, the possibility of a tumor inhibition effect on cellular metabolism that increased the apoptosis of tumor cells was analyzed in vivo. The anticancer effect of GGMPN was then evaluated by investigating the extent of apoptosis induction by TUNEL staining in vivo (Figure 6C). In agreement with in vitro signal expression results, HepG2 tumors considerably induced apoptosis protein expression in mice treated with GGMPN. We also found that the Au concentration in the nude mice injected with GGMPN was significantly higher in tumor tissues than in the other groups (Figure 6D). In summary, these results demonstrated that the delivery of miR-122 by the multifunctional effect of GGMPN provided a novel and effective strategy to induce apoptosis and inhibit the growth of tumor cells.

## Conclusions

Taken together, GGMPN has the following advantages: (1) due to the high proportion of specific surface area of graphene, the drug loading capacity of the nanocomposite is greatly improved; (2) the properties of graphene and gold nanoparticles result in low toxicity; (3) bound P-gp antibody and FA has preferred targeting ability; (4) the nano-composite can increase the permeability of a drug-resistant cancer cell membrane, promote the gold particle uptake by the cancer cells and enhance intracellular drug accumulation; (5) the nano-composites have good properties to promote drug-resistant cancer cell apoptosis; (6) the nano-composites attach to tumor cells, resulting in efficient photothermal therapy for killing cancer cells; and (7) the nano-composites allow specific fluorescent bio-marking of the tumors.

## Abbreviations

GGMPN: Monoclonal P-glycoprotein (P-gp) antibodies, folic acid (FA) and miR-122 loaded gold nanoparticles on graphene nanocomposites
MTT: 3-(4,5-dimethylthiazol-2-yl)-2,5-diphenyl tetrazolium bromide
PARP: Proteolytic cleavage of poly-(ADP-ribose) polymerase
TUNEL: Terminal deoxynucleotidyl transferase-mediated dUTP nick end-labeling
